# Basic principles of the virulence of *Cryptococcus*

**DOI:** 10.1080/21505594.2019.1614383

**Published:** 2019-05-23

**Authors:** Oscar Zaragoza

**Affiliations:** Mycology Reference Laboratory National Centre for Microbiology, Instituto de Salud Carlos III Carretera Majadahonda-Pozuelo, Madrid, Spain

**Keywords:** Cryptococcus, capsule, melanin, virulence, titan cell

## Abstract

Among fungal pathogens, *Cryptococcus neoformans* has gained great importance among the scientific community of several reasons. This fungus is the causative agent of cryptococcosis, a disease mainly associated to HIV immunosuppression and characterized by the appearance of meningoencephalitis. Cryptococcal meningitis is responsible for hundreds of thousands of deaths every year. Research of the pathogenesis and virulence mechanisms of this pathogen has focused on three main different areas: Adaptation to the host environment (nutrients, pH, and free radicals), mechanism of immune evasion (which include phenotypic variations and the ability to behave as a facultative intracellular pathogen), and production of virulence factors. *Cryptococcus neoformans* has two phenotypic characteristics, the capsule and synthesis of melanin that have a profound effect in the virulence of the yeast because they both have protective effects and induce host damage as virulence factors. Finally, the mechanisms that result in dissemination and brain invasion are also of key importance to understand cryptococcal disease. In this review, I will provide a brief overview of the main mechanisms that makes *C. neoformans* a pathogen in susceptible patients.

**Abbreviations**: RNS: reactive nitrogen species; BBB: brain blood barrier; GXM: glucuronoxylomannan; GXMGal: glucuronoxylomannogalactan

Microscopic fungi comprise a large number of microorganisms of very different characteristics. Among them, a few can cause disease in humans, especially among immunocompromised patients [1]. Opportunistic invasive fungal diseases have become a problem of concern for the national health systems worldwide due to their high associated mortality and economical cost [2,3].

The main human fungal pathogens belong to the genera *Candida, Aspergillus,* and *Cryptococcus*. All of them are cause disease in immunosuppressed patients, so they are considered opportunistic pathogens. The mechanisms involved in invasive fungal disease are different and involved both fungal and host elements. Some of pathogenic mechanisms are shared by all the fungal pathogens, such as the ability to grow at physiological temperature, resistance to the immune challenges (such as free radicals), and others. But the case of *Cryptococcus* offers an excellent model to investigate fungal pathogenesis for several reasons. First, it is a pathogen of concern because it has a high prevalence in certain geographical regions. But in addition, this yeast has developed some pathogenic mechanisms and acquired some virulence traits that make it an unique fungal pathogen. In this review, I will summarize the main reasons why *Cryptococcus* is a different fungal pathogen.

## Epidemiology, incidence and disease

*Cryptococcus* spp are basidiomycetes yeasts, which is a different characteristic from most human pathogenic fungi, which are ascomycetes. Its metabolism is mainly respiratory, so its growth is highly dependent on the presence of oxygen [4]. From a structural point of view, *Cryptococcus* spp present a unique phenotypic trait, which is the presence of a capsule that surrounds the cell body.

*Cryptococcus* spp are widely found in the environment, but there are two species that have been associated to disease in humans: *Cryptococcus neoformans* and *Cryptococcus gattii* [5]. These two yeasts are very closely related, but present differences in the epidemiology and disease in humans. The most prevalent is *C. neoformans*, which behaves as opportunistic pathogen in immunosuppressed patients. *Cryptococcus gattii*, in contrast, has a lower incidence, but can cause disease in immunocompetent patients [5]. Most studies about virulence have focused on *Cryptococcus neoformans*, and for this reason, this review will focus mainly on this yeast.

*Cryptococcus neoformans* has been known as causative agent of disease since the 19^th^ century [5]. However, its incidence raised significantly at the end of the 20^th^ century, associated with the emergence of HIV infection, and it was estimated that it could affect around 10% of AIDS patients. The introduction of the antiretroviral therapy has controlled the incidence of cryptococcosis in developed countries, but it is still a major concern in developing areas. Globally, it is estimated that it causes around 180,000 deaths in Subsaharian Africa, although it has also an important incidence in Asia and South America [6,7].

The infection is acquired by inhalation of spores or desiccated conidia from the environment, and the pathogen first colonizes the lung [4]. The immune response of this organ is very specific because it is continuously exposed to exogenous particles [8], and in fact, it is very effective at controlling the dissemination of the cryptococcal cells. However, in immunosuppressed patients, in particular those defective in CD4 T cells, *C. neoformans* can replicate and disseminate through the organism. The most characteristic clinical outcome appears when it reaches the brain, where it causes meningoencephalitis. This disease is very serious and has a high mortality associated (around 20–50% of affected patients).

## *Cryptococcus* as environmental pathogen

When compared to other fungal pathogens, *C. neoformans* is very characteristic in the infection route and in the disease caused. For examples, other yeasts, such as *Candida* spp, are rarely acquired by inhalation and *C. neoformans* resembles more the infection caused by filamentous fungi or primary fungal pathogens, such as *Histoplasma capsulatum. Cryptococcus neoformans* has developed some virulence mechanisms that allow the survival in the lung and dissemination to the brain. Interestingly, some of them are also used to infect and cause disease in environmental host [9]. This yeast has a worldwide distribution and it can be disseminated using some birds as carriers [10]. It is believed that the continuous exposure to environmental stress, such as temperature fluctuations and dehydration has selected cryptococcal strains with a higher fitness in mammalian individuals. Furthermore, it has the ability to infect a large number or organisms, such as amoebas, flies, nematodes, Lepidoptera, and even plants as *Arabidopsis thaliana* [11–15]. Even in the case of mammals, there are reports of infections in a wide range of animals, such as koalas, dolphins, and cats [16,17]. These multiples interactions are believed to be important for the virulence of *C. neoformans*, because the mechanisms that allow the fungal survival after interaction with these different hosts have selected multiple traits that can be used to adapt and cause disease in humans. Maybe the phenomenon that is best characterized is its ability to survive after the interaction with environmental predators, such as amoeba [18,19]. This cryptococcal ability is very similar to the behavior during the interaction with mammalian phagocytic cells.

## Cryptococcal pathogenesis

Infection by *C. neoformans* is believed to occur through inhalation infective particles. In this sense, it has been shown that cryptococcal spores can germinate and cause disease [20–22]. After inhalation, *C. neoformans* must survive in the lung and evade the immune response of this organ, which has an specialized immune response [8]. The alveoli are covered by a layer of alveolar macrophages, which are in charge of the phagocytosis and elimination of these challenges. In addition, lymphocytes (such as CD4, CD8) and other myeloid cells (dendritic cells) are also recruited in this organ. The lung also contains the surfactant, which main function is to maintain surface tension of the pleura during the respiration process. In addition, some proteins from the surfactant have antimicrobial properties.

In this complex environment, cryptococcal virulence is dependent on three main types of processes: Adaptation to the host environment, mechanisms of immune evasion, and production of true virulence factors. Although these three processes should be applied to most fungal pathogens, *C. neoforman*s is unique because it has developed many different types for adapting to the host, evade the immune response, and produce true virulence factors [23]. In the next sections, we will provide a brief overview on the main process that make *C. neoformans* a unique fungal pathogen.

### Adaptation mechanisms to host conditions

As any other microbial pathogen, survival in the host involves the induction of adaptation mechanisms to physiological temperature, different sources of nutrients, pH, and oxidative stress (see seminal review in Ref. [24]). For adaptation to these conditions, *C. neoformans* induces multiple metabolic rearrangements and activation of signaling pathways (such as MAPKs [25]). However, some aspects of adaptation are of great interest. One is the mechanisms that allow growth at our physiological temperature. Environmental fungi normally do not tolerate high temperatures, including the one from our body [26]. And in fact, most pathogenic fungi are those able to replicate at 37ºC. This indicates that this adaptation is most probably one of the main factors to become a pathogen in an immunosuppressed host [27]. Some of the processes required for growth at higher temperatures involve antioxidant responses, trehalose accumulation, and activation of the different signaling pathways [28–31].

Another adaptation process required for survival *in vivo* are those needed for the uptake of some limiting, but essential nutrients such as metals that are required for many metabolic reactions [32]. Among them, the role of iron in cryptococcal virulence is one of the most studied [33]. This metal is required for the proper activity of many enzymes in the cell, and both yeast and host cells have developed mechanisms to efficiently compete and obtain it from tissues and body fluids. In the case of *C. neoformans*, several proteins are required for efficient iron uptake, such as iron permeases, ferroxidases, and the glycoprotein Cig1, which is chelator of heme groups [34–36].

The pH is another factor can change, not only in the host, but also in the environment, and for this reason, the ability to adapt to different pHs is also required to survive during infection [37]. One of the main proteins required for adaptation to neutral pH is Rim101, which encodes a transcription factor that responds to alkaline pH [38]. Interestingly, this protein also regulates other important features required for virulence, such as cell wall integrity and capsule production [39–41].

Another process required for survival during infection involves the adaptation to free radicals. One of the main mechanisms elicited by the immune response to induce pathogen killing is oxidative and nitrosative stress. In *C. neoformans*, adaptation to reactive oxygen species depends mainly based on gluthatione and thioredoxin [42–44] and mannitol [45]. Regarding resistance to reactive nitrogen species (RNS), several proteins have been involved in this process. Resistance to nitrosative stress depends on the reductive power (production of NADPH) of the cells and it has been shown that isocytrate dehydrogenase is important for this process. This enzyme catalyzes the conversion of isocytrate to α-ketoglutarate, a reaction that also produces NADPH. Absence of isocytrate dehydrogenase results in increased susceptibility to RNS due to lower production of NADPH and mitochondrial disorders [46]. In agreement, a favohemoglobin denitrosylase (whose activity depends on NADPH) is important for detoxification of RNS [47,48].

### Virulence factors

The definition of virulence factors is complex and very heterogeneous according to the source, but there is consensus that they can be defined as those elements of a pathogen that can cause damage in the host [49]. Following this definition, *C. neoformans* produces several degrading enzymes, such as proteases and lipases as virulence factors. These enzymes are also produced by other microbial pathogens, including fungi and bacteria [50–52]. In the case of *C. neoformans*, there is another degrading enzyme, urease, which also plays a role during infection. Urease catalyzes the degradation urea into CO_2_ and ammonia, and is required for nitrogen utilization in multiple organisms. *Cryptococcus neoformans* produces very high amounts of urease, and it presence has been used as a diagnostic tool for cryptococcosis [53]. Urease is considered a virulence factor. Absence of this enzyme results in a fitness defect at slightly basic pH [54]. During infection, urease is required for brain invasion [55]. Urease promotes sequestration of cryptococcal cells at microcapillary vessels [56], and it has been hypothesized that ammonia promotes adhesion of *C. neoformans* either by increasing the expression of adhesins on the endothelia or by a direct toxic effect on the integrity tight junctions of the brain blood barrier (BBB) that would facilitate the brain invasion [56,57]. In agreement, proteins required for urease activity are also defective in brain invasion [58].

However, *C. neoformans* is well known because it expresses two clear virulence factors that interfere with the host immune systems, which are the capsule and melanin. Interestingly, these two components have a dual role, not only as virulence factors, but also as protective elements against some attacks of the immune response.

#### The polysaccharide capsule

The capsule is the structure that has been characterized in *C. neoformans* in most detail. It is mainly composed of two types of polysaccharides: glucuronoxylomannan (GXM) and glucuronoxylomannogalactan (GXMGal) [59–62]. There are other minor components, which are mannoproteins and chitin-like structures [63–65], but their function and role in capsule structure is still unknown.

Capsule structure is complex, and interestingly, it varies according to the environmental conditions. It is organized in branched fibers that attach to the cell wall and bind to each other through non covalent bonds [66,67].

Capsule synthesis is one of the topics of great interest in the biology of this pathogen (see reviews in Refs. [68–71]). Many of the proteins required for capsule synthesis are glycosidases that catalyze the interconversion of the different sugars that are bound to form the polysaccharide fibers (see reviews in Refs. [72–76]). However, there are still many aspects of capsule synthesis that remain unknown. In the last years, there is increasing evidence that the polysaccharide components are synthesized in the ER and exported inside small vesicles [77]. The characterization of these vesicles has demonstrated that they contain, not only the capsular polysaccharide, but also other virulence factors that contribute through disease [78], and for example, they can influence the virulence of *C. neoformans* in macrophages [79].

The capsule plays an important role in the virulence of *C. neoformans*. However, it is not required for the regular life of the yeast since acapsular mutants can survive and replicate *in vitro*, but it absence results in defects in virulence [80]. This is in part due to two main reasons. First, the capsule has some protective effects during infection. It has been shown that the capsular polysaccharide inhibits phagocytosis [81,82]. The most plausible explanation for this avoidance is that most epitopes that bind to the macrophage receptors (mainly cell wall components) are at the cell wall, and the presence of the capsule “hides” them from the phagocytic cell.

The capsule also confers protection against some stress factors, such as dehydration and free radicals [83–85].

The second function of the capsule during infection is its role as a true virulence factor. Many different studies have demonstrated that the capsular polysaccharides are secreted and have multiple effects on the host immune response (see reviews in Refs. [86,87]). Most data has been focused on GXM because it is the most abundant component of the capsule. This polysaccharide can inhibit neutrophil migration in different ways. GXM inhibits the exit of leukocytes from the blood vessels because it exhibits chemoattracting properties [88] and decreases the expression of chemokine receptors [89]. In addition, it inhibits the binding of leukocytes to endothelium by inducing L-selectin and E-cadherin shedding from neutrophils [89–91] and also by binding to CD18 [92].

Early studies demonstrated that infection with capsular polysaccharide also produces an immunological unresponsiveness, which is mainly manifested by an inhibition in the production of antibodies [93,94]. Later on, many different deleterious effects have been attributed to these polysaccharides. Among others, both GXM and GalGXM alter the cytokine production and affects maturation of dendritic cells and antigen presentation [95–99]. Another interesting mechanism involved in immune unresponsiveness is the effect of the capsular polysaccharides on the viability of some leukocytes. It has been shown that both GXM and GalGXM are potent inducers of apoptosis [100–103], which adds another role for the capsule as virulence factor.

The capsule can also interfere with elements of the host innate immune system. The polysaccharide inhibits the agglutination of cryptococcal cells by the surfactant protein SP-D. It is also noteworthy that SP-D^−/−^ mice are paradoxically more resistant to infection [104,105], which suggests that *C. neoformans* has developed a mechanism to induce protective responses through some of the surfactant proteins.

#### Melanin

The other typical phenotypic feature of *C. neoformans* is the accumulation of melanin, which is a dark pigment abundantly found in animals and fungi. In the case of *C. neoformans*, melanin is only produced in the presence of exogenous compounds, mainly diphenolic compounds as L-DOPA [106,107]. Melanin synthesis depends on an enzyme, diphenol oxidase, encoded by two genes, *LAC1* and *LAC2*, being Lac1 the main protein involved in the production of this pigment [108].

Melanin plays a profound effect in the biology and virulence of *C. neoformans*. Mutants unable to melanize present a significant reduction in their virulence [109,110]. It is well known that this pigment confers resistance to multiple stress factors, such as free radicals, ionizing radiation, and heat [111–113]. It can also bind and decrease the susceptibility to antifungal drugs [114,115]. Interestingly, a recent study has demonstrated that cryptococcal melanin enhances heat capture and contributes in this way to growth at low temperature [116].

In addition to its protective role, melanin has been involved in other processes required for virulence, and for this reason, is considered a true virulence factor too. Melanin seems to play a key role in the dissemination from the lung to the brain [117]. Melanization also changes the host cytokine production [118] and protects against macrophages [119]. The role of melanin as virulence factor has been established from the study of melanin particles (denominated melanin ghosts). Injection of mice with purified cryptococcal melanin [120] results in the formation of granulomas in the spleen, lung, and liver, indicating that this pigment can induce pro-inflammatory responses that alter the host immune response.

### Mechanisms for evasion of host immune response

*Cryptococcus neoformans* is a very particular yeast because it has developed some specific mechanisms that allow the evasion of some of the main attacks of the immune response, and in consequence, persist in tissues and organs for long periods.

#### Phagocytosis avoidance and intracellular pathogenesis

One of the areas characterized in most detail is the interaction of *C. neoformans* with phagocytic cells. After inhalation, spores and infectious particles have to face a layer of alveolar macrophages, which poses the first defense line in this organ. Although the capsule inhibits phagocytosis (see above), cryptococcal cells can in fact be phagocytosed in the presence of opsonins, mainly complement proteins and circulating antibodies. Even inside the macrophage, *C. neoformans* can survive and avoid killing [121,122]. This yeast is, in consequence, considered a facultative intracellular fungal pathogen because it can replicate inside the phagocytic cell and live in this niche for long time periods. At the moment, there are several responses that allow the intracellular survival. Cryptococcal cells inhibit the full acidification of the phagolysosome [123], which results is lower antimicrobial activity in this compartment. This inhibition is partially mediated by the activity of urease, which degrades urea into CO_2_ and ammonia, which neutralizes the acidic pH of the phagolysosome [54]. In parallel, the phagolysosomes become leaky, which may also affect the functionality of the phagolysosome [122]. In agreement, it has been recently shown that interference of phagosome acidification of cryptococcal-infected macrophages correlates with higher replication of the fungal cells *in vivo* [124]. Finally, the capsule of *C. neoformans* has antioxidant properties [84], which also contributes to resistance to the free radicals produced in the phagolysosome. All these mechanisms are thought to be essential for the survival of cryptococcal cells in macrophages.

Another of the interesting research topics in the cryptococcal field is the outcome of yeast cells in the macrophages, and different processes might occur. For example, massive fungal replication might result in macrophage explosion. But yeast cells can be expelled from the macrophage through a process that does not affect the viability of the fungus nor the phagocytic cell. This process has been denominated as vomocytosis or non-lytic exocytosis [125,126]. Similarly, the cryptococcal cells can be transferred between macrophages [127,128]. Even depending in the activation state of the macrophage, the yeast cells can be efficiently destroyed and eliminated. And to make the situation more complex, several mechanisms might occur at the same time. For example, as observed in Supplemental Video 1, some yeast cells can replicate within a macrophage at the same time that other cryptococcal cells are attacked and eliminated in the same phagocytic cell.

In summary, the outcome of the interaction between *C. neoformans* and phagocytic cells is very complex, and many situations are possible. At the moment, we know some the factors that influence the fate of cryptococcal cells in macrophages, so more work is required to fully characterize this process, because it is believed that the result of this interaction is important to understand many aspects of the virulence of *C. neoformans*.

#### Capsular phenotypic variations

*Cryptococcus neoformans* has developed other mechanisms that contribute to the evasion of the host immune system, which are related to changes in some phenotypic features at the capsule and in the cell size of the yeast.

The capsule of *C. neoformans* is very dynamic, and it can undergo different rearrangements during infection (see reviews in Refs. [129,130]. In particular, there are three typical changes that have been described in this structure. First, the capsule can change in size. Early studies described that several factors can induce capsule growth in vitro, such as CO_2_, iron limitation, mammalian serum, and nutrient limitation at basic pH [131–134]. More importantly, capsular enlargement is one of the first responses induced by *C. neoformans* in different types of hosts, including mammals, amoebas, and insects [135–137]. This process is important for the survival of the yeast during infection and to avoid the immune response. It has been shown that cells with enlarged capsule are more resistant to oxidative stress, antimicrobial peptides, and antifungals [84]. Furthermore, capsule growth also impairs phagocytosis mediated by complement [138]. In agreement, there is a correlation between the size of the capsule of the yeasts in the CSF and the intracranial pressure of patients with meningoencephalitis [139], suggesting that this process participates in the disease caused by this pathogen.

The capsule of the *C. neoformans* can also change its epitope structure both in vitro and during infection [140,141]. The importance of this process in the virulence has not been fully elucidated, but it has been correlated with the dissemination of the yeasts to the brain during infection [141]. Changes in the structure could also contribute to “hide” the fungal cells from elements of the immune system, such as circulating antibodies.

The third typical capsular change is related to the amount of polysaccharide and the density of this structure [142,143]. In parallel to capsule growth, the density of the polysaccharide capsule increases gradually during time *in vitro* [143]. This process correlates with the age of the cell. And in agreement, fungal cells isolated from *in vivo* have a more dense capsule due to a higher accumulation of polysaccharide fibers [142]. Increases in capsule density are believed to contribute to immune evasion by hindering the penetration of molecules, such as antimicrobial peptides, and by inducing protection against other stress factors, such as free radicals.

#### Cryptococcal morphogenesis and immune evasion

A characteristic feature of fungi is the ability to grow in different morphological forms. The process that is more widely described is the formation of filaments, such as hyphae and pseudohyphae. In the case of fungi that can cause disease, the induction of morphological changes contributes to some important virulence mechanisms, such adhesion and penetration into mucosae and tissues, and dissemination through the body [144–146]. Morphogenesis in *C. neoformans* is very peculiar and characteristic. This fungus can form pseudohyphae [147,148], but their exact role during infection remains to be fully understood. In contrast, there is a very unique and typical change induced *in vivo*, which involves a significant increase in the cell size. While cryptococcal cells *in vitro* have a size around 4–6 microns, in vivo the cell body can reach up to 40–50 microns [135,149,150]. This change is even more dramatic if the size of the capsule is considered, finding yeast cells of a total size that can reach up to 70–100 microns. These cells have been denominated as “titan cells” [151]. Due to their large size, the appearance of these cells results in a fungal population that cannot be easily eliminated by the immune system. Titan cells cannot be phagocytosed, are more resistant to stress factors and in consequence, contribute to fungal persistence during long time periods [149,150,152]. But there is also evidence that titan cells can also actively contribute to some important virulence mechanisms. For example, although due to their size these cells cannot invade nor penetrate biological barriers, titan cells can replicate and originate a progeny of daughter cells of regular size that can disseminate to other organs [153]. Titan cells can also impair the phagocytosis of yeast cells of regular size. Furthermore, the appearance of titan cells also seems to induce changes in the host immune response and cause a Th2 polarization [154,155], which are associated with non-protective responses during cryptococcosis.

Research on titan cells has been limited due to the difficulty to obtain *them in vitro*, and most knowledge about this transition has been obtained from the characterization of cells isolated from infected mice. However, recently, three different groups have identified conditions that mimic *in vitro* the formation of titan cells [156–158]. These conditions include incubation in nutrient limited media, serum, low cell density, and oxygen limitation. These articles have highlighted new conditions that induce titanization in *C. neoformans*. Cellular growth is enhanced when the cultures are inoculated with a low cell density, suggesting that the formation of titan cells is negatively regulated by quorum sensing molecules. In agreement, addition of Qsp1 (which is a short peptide that regulates QS in this pathogen [159,160]) inhibits titan cells formation [156,157], and deletion of the encoding gene results in an increase in cell size during hypoxia [156]. Another factor that induces titanization is addition of serum [157,158]. The central pathway involved in the development of titan cells depends on cAMP [150,157,158,161,162]. Several upstream and downstream elements on this pathway have been shown to regulate titanization in *C. neoformans*. Among them, the Ste3α pheromone receptor and the G-protein-coupled receptor Gpr5 are needed for titan cell formation, and both of them activate cAMP synthesis through the G-protein Gpa1 [149,161]. Furthermore, the Rim101 transcription factor, which is regulated by cAMP, is also required for titanization [161].

In summary, the discovery of *in vitro* conditions that produce the appearance of titan cells will greatly contribute in the future to understand the exact role of these cells during infection.

### Dissemination to the brain

Despite survival in the lung is important in the virulence of *C. neoformans*, the dissemination from this organ through the organism and in particular to the brain. For still unknown reasons, this fungus has special tropism for the brain, where it causes meningoencephalitis. For this reason, the mechanisms that cause the dissemination and invasion of the BBB and later survival in the brain have been a priority in cryptococcal research [163,164]. And in fact, there is evidence that *C. neoformans* can invade the brain by different and concomitant mechanisms.

At the moment, three different ways of dissemination and BBB invasion have been proposed. Cryptococcal cells can bind to the luminal side of the BBB and be endocytosed by the endothelial cells [165,166]. Binding and invasion depends on host elements (such as CD44 and annexin A2 [167,168]) and cryptococcal factors, such as urease, phospholipase B, secretion of hyaluronic acid, and some metalloproteases [56,169,170,171].

The second mechanisms involves damage of the tight junctions of the BBB and cross of the cryptococcal cells between the endothelial cells (paracellular passage) [56,141,168].

Finally, the third mechanism is related to the ability of *C. neoformans* to survive in phagocytic cells. The intracellular pathogenesis of *C. neoformans* can provide not only a mechanism of immune evasion, but also a way to disseminate through the organism “hidden” inside infected phagocytic cells, a process denominated as the “Trojan horse” dissemination mechanism. In the last years, different groups have accumulated strong evidence that support the Trojan horse hypothesis. Depletion of macrophages results in reduced fungal burden in the brain, and injection of mice with macrophages infected *ex vivo* with *C. neoformans* increases the number of yeast cells in this organ [172]. Furthermore, the Trojan Horse migration process has been also demonstrated *in vitro* using BBB models in transwell plates [173,174]. This migration is enhanced by some immune mediators, such as MCP-1, IFN-γ, and TNF-γ. Interestingly, mutants that are defective in dissemination can cross in vitro generated BBB as wild type strains, supporting the role of the Trojan Horse mechanism in cryptococcal dissemination [174].

## Conclusions

*Cryptococcus neoformans* was first described as pathogen at the end of the 19^th^ century, although the characterization of its virulence mechanisms has been mainly studied in the last four decades. Despite the great effort invested by the scientific community in this pathogen, there are still great challenges to manage and decrease its impact. An example is the characterization of the capsule. This is the structure best characterized in this yeast, but we still do not know how it is synthesized in detail. A similar situation occurs with other virulence traits, such as intracellular pathogenesis. This is a characteristic cryptococcal feature, but its full contribution to virulence still remains unknown. A key process of cryptococcal disease is the dissemination of the yeasts to the brain. In this topic, there have been importance advances, but there is still a need to develop strategies to inhibit this step and brain invasion. The current antifungal therapy for cryptococcal disease is very limited, since echinocandins are not active against *C. neoformans*, and treatment is based on amphotericin B and fluconazole. Even though Amphotericin B is very active, its toxicity limits its use. There are some liposomal formulations that reduce the negative effects of this antifungal, but their high prices difficult their application in developing countries. For these reasons, future research is still needed to understand the virulence of this fungal pathogen.
