# Supplementary material for: Basic principles of the virulence of Cryptococcus
Source: Virulence. 2019 May 23;10(1):490–501. doi: 10.1080/21505594.2019.1614383 (PMC6550552; doi:10.1080/21505594.2019.1614383)
Supplement: Supplemental Material [file kvir-10-01-1614383-s002.docx]

**Legend for supplemental figure 1. Heterogeneity of *C. neoformans* behavior inside phagocytic cells.**

GFP-labeled cryptococcal cells were phagocytosed by RAW264.7 macrophages and intracellular behavior was followed by real time microscopy. Each second of the video corresponds to 30 m of real time. Black arrow denotes cryptococcal cells that replicate inside the macrophage, and white arrows highlight yeast cells that are killed and removed by the phagocytic cell.
